# Supplementary material for: Inclusion of Metabolic Tumor Volume in Prognostic Models of Bone and Soft Tissue Sarcoma Increases the Prognostic Value
Source: Cancers (Basel). 2023 Jan 28;15(3):816. doi: 10.3390/cancers15030816 (PMC9913525; doi:10.3390/cancers15030816)
Supplement: Supplementary file 1 [file cancers-15-00816-s001.zip › cancers-2196778-supplementary.pdf]

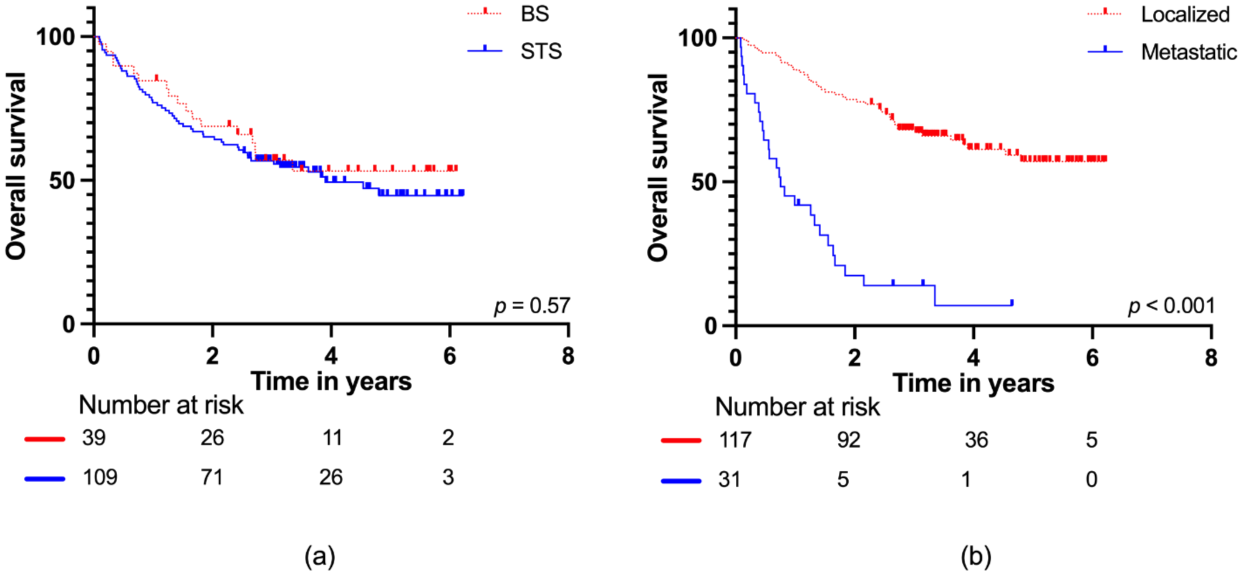

Figure S1. Kaplan–Meier survival curves with number at risk at baseline, two, four, and six years for (**a**) BS vs. STS and (**b**) metastatic vs. localized disease.

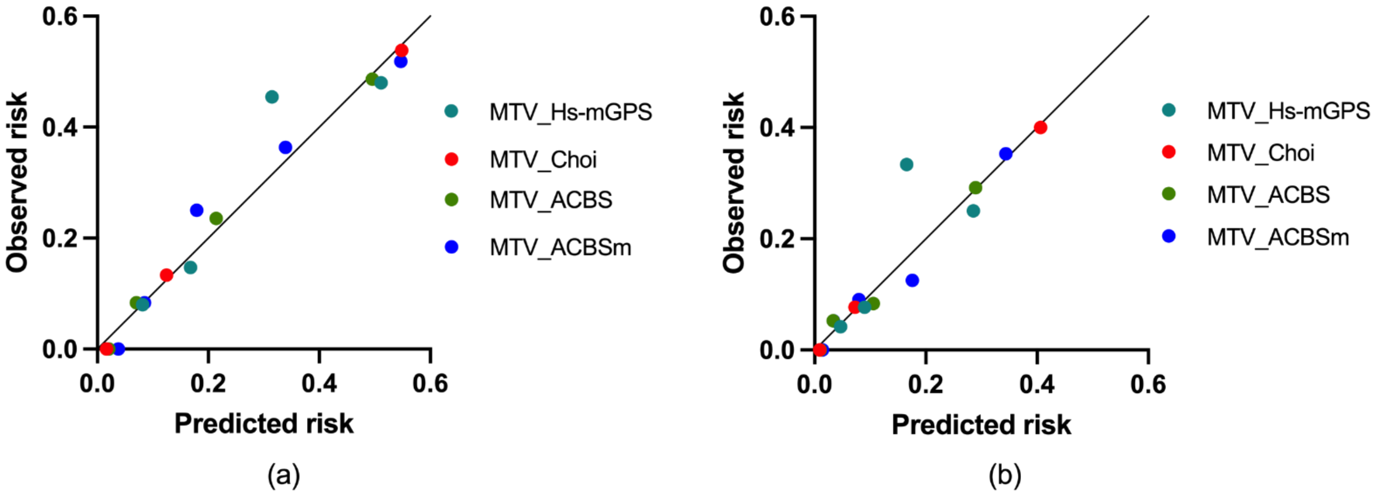

Figure S2. Calibration plots for one-year survival in (**a**) all patients and (**b**) patients with localized disease.

Table S1. New combined prognostic scores.

**New combined prognostic scores**	
MTV_HS-mGPS<br>Parameters included: MTV2.5, CRP, albumin	
0	All normal, albumin indifferent
1	One high/elevated, albumin indifferent
2	MTV2.5 and CRP high/elevated, albumin normal
3	All high/elevated
MTV_Choi<br>Parameters included: MTV2.5, ESR, CRP, NLR	
0	All normal
1	Two or three high/elevated
2	All high/elevated
MTV_ACBS<br>Parameters included: MTV2.5, albumin, haemoglobin, CRP, neutrophils, lymphocytes	
0	All normal
1	One high/elevated
2	Two high/elevated
3	Three or more high/elevated
MTV_ACBSm<br>Parameters included: MTV2.5, albumin, haemoglobin, CRP, neutrophils, lymphocytes, Na+	
0	All normal
1	One high/elevated
2	Two high/elevated
3	Three or more high/elevated

Table S2. Hazard ratios for overall survival in current predictive models.

Score	All Patients				Localized Disease			
	n (%)	Events	HR (95% CI)	*p*-Value	*n* (%)	Events	HR (95% CI)	*p*-Value
HS-mGPS								
0	49 (52)	19	1		41(57)	12	1	
1	18 (19)	9	1.43 (0.65–3.16)	0.38	12 (17)	5	1.54 (0.54–4.38)	0.42
2	28 (29)	19	2.72 (1.43–5.14)	<0.01 *	19 (26)	10	2.37 (1.02–5.49)	0.04 *
Total	95 (100)	47			72 (100)	27		
Choi								
0	16 (31)	6	1		14 (33)	4	1	
1	35 (69)	20	2.07 (0.83–5.17)	0.12	29 (67)	14	2.09 (0.69–3.70)	0.19
Total	51 (100)	26			43 (100)	18		
ACBS								
0	28 (30)	8	1		25 (35)	6	1	
1	26 (27)	11	1.69 (0.68–4.20)	0.26	19 (27)	5	1.13 (0.34–3.70)	0.84
2	40 (43)	27	3.76 (1.70–8.29)	<0.01 *	27 (38)	15	3.10 (1.20–7.99)	0.02 *
Total	94 (100)	46			71 (100)	26		
ACBSm								
0	28 (30)	8	1		25 (35)	6	1	
1	25 (26)	11	1.77 (0.71–4.40)	0.22	18 (25)	5	1.19 (0.36–3.91)	0.77
2	12 (13)	6	2.19 (0.76–6.33)	0.15	9 (13)	3	1.50 (0.37–5.99)	0.57
3	29 (31)	21	4.42 (1.95–10.03)	<0.01 *	19 (27)	12	3.90 (1.46–10.40)	<0.01 *
Total	94 (100)	46			71 (100)	26		

* indicates statistical significance

Table S3. AIC and C-index for PET parameters, current prognostic scores, and combined models.

		MTV2.5		SUVmax		TLG2.5	
	n	**AIC**	**C-Index**	**AIC**	**C-Index**	**AIC**	**C-Index**
PET	148	634	0.66	637	0.65	630	0.67
HS-mGPS	95	390	0.64	390	0.64	390	0.64
PET + HS-mGPS	95	377	0.71	386	0.68	382	0.69
Choi	51	188	0.59	188	0.59	188	0.59
PET + Choi	51	184	0.66	188	0.62	186	0.64
ACBS	94	376	0.67	376	0.67	376	0.67
PET + ACBS	94	365	0.72	374	0.70	371	0.70
ACBSm	94	377	0.68	377	0.68	377	0.68
PET + ACBSm	94	367	0.73	375	0.70	372	0.70
PET	95	376	0.65	389	0.61	380	0.65
HS-mGPS	95	390	0.64	390	0.64	390	0.64
PET + HS-mGPS	95	377	0.71	386	0.68	382	0.69
PET	51	185	0.60	189	0.56	186	0.61
Choi	51	188	0.59	188	0.59	188	0.59
PET + Choi	51	184	0.66	188	0.62	186	0.64
PET	94	368	0.66	380	0.61	371	0.65
ACBS	94	376	0.67	376	0.67	376	0.67
PET + ACBS	94	365	0.72	374	0.70	371	0.70
PET	94	368	0.66	380	0.61	371	0.65
ACBSm	94	377	0.68	377	0.68	377	0.68
PET + ACBSm	94	367	0.73	375	0.70	372	0.70
PET	51	185	0.60	189	0.56	186	0.61
HS-mGPS	51	190	0.60	190	0.60	190	0.60
PET + HS-mGPS	51	187	0.65	191	0.63	189	0.63
Choi	51	188	0.59	188	0.59	188	0.59
PET + Choi	51	184	0.66	188	0.62	186	0.64
ACBS	51	187	0.63	187	0.63	187	0.63
PET + ACBS	51	185	0.67	188	0.66	187	0.68
ACBSm	51	186	0.65	186	0.65	186	0.65
PET + ACBSm	51	185	0.70	188	0.67	188	0.69
